# Human Circadian Rhythm Through the Lens of GIScience: A Systematic Review

**DOI:** 10.21203/rs.3.rs-9876764/v1

**Published:** 2026-06-18

**Authors:** Mingzheng Yang, Lei Zou, Esther Doria, Joynal Abedin, Binbin Lin, Heng Cai, Wanhe Li

**Affiliations:** 1. Department of Geography, Texas A&M University, College Station, the United States; 2. Department of Geography, Texas A&M University, College Station, the United States; 3. Department of Biology, Texas A&M University, College Station, the United States; 4. Department of Geography, Ohio University, Athens, the United States; 5. Department of Geography, Texas A&M University, College Station, the United States; 6. Department of Geography, Texas A&M University, College Station, the United States; 7. Department of Biology, Texas A&M University, College Station, the United States; 8. Center for Biological Clocks Research, College Station, the United States; Texas A&M University

**Keywords:** Circadian Rhythm, GIScience, Spatial-Temporal Analysis, Sleep Health, Social-Environmental Factors

## Abstract

Geographic Information Science (GIScience) offers promising data and tools to innovatively capture and analyze human circadian rhythms across space and time at unprecedented scales, yet its potential remains underexplored. This systematic investigation examines 60 studies published between 2001 and 2024 that applied GIScience to investigate circadian rhythm patterns and their associations with social-environmental factors. Most studies relied on survey or sensor-based data, with limited use of emerging sources such as social media, mobility, web applications, and remote sensing. We found that previous research largely emphasized individual- and population-level monitoring, particularly at the city scale with minute-level temporal resolutions. Traditional statistical approaches dominated, while spatial analysis and advanced spatial-temporal modeling techniques were rarely applied. Findings highlight that both geographic attributes (e.g., latitude, longitude, altitude) and social-environmental conditions (e.g., light, noise, socioeconomic status) exert spatially heterogeneous influences on circadian rhythms, underscoring the need for greater integration of GIScience data and methods into circadian research.

## Introduction

The term ‘circadian rhythm’ originates from a physiological concept (Mills, 1966), referring to the endogenous, approximately 24-hour biological cycle that regulates human external behaviors and internal molecular processes (Czeisler et al., 1999). External disturbances from social and environmental changes can induce significant circadian rhythm disruptions. One of the most important environmental factors is light exposure. Inappropriate timing of light exposure can lead to phase shifts in circadian rhythms (Rust et al., 2011). For instance, exposure to light at night, a common phenomenon in urban environments, can delay the circadian phase and suppress melatonin production, a hormone critical for sleep regulation (Gooley et al., 2011). In addition to environmental factors, societal changes and conditions can cause social jet lag, which is the misalignment between biological time and socially imposed schedules such as early school or work start times. This phenomenon is especially pronounced in adolescents and young adults, whose natural circadian preference (chronotype) tends to be later (Roenneberg et al., 2007).

Although circadian rhythms are influenced by a complex interplay of social-environmental factors (Leypunskiy et al., 2018; Martins et al., 2020), most existing biological studies overlooked their geographical patterns and disparities. There is a lack of research investigating the underlying mechanisms on how different social and environmental contexts shape the geospatial variations of circadian rhythms. Identifying geographic features of circadian rhythms and their correlation with geospatially varying social-environmental factors are essential for recognizing areas and populations at high risk of circadian rhythm disorders, identifying local determinants and mechanisms, and developing place-specific interventions to address their root causes.

Advances in Geographic Information Science (GIScience) can provide powerful tools to address this gap. Geographic Information Systems (GIS) enable the integration of diverse datasets, including environmental exposures, socioeconomic conditions, remote sensing data, and health surveys, to analyze spatial variations in circadian rhythm changes (Luan and Law, 2014). Spatial analysis methods, such as spatial autocorrelation, hotspot detection, and clustering, help identify geographic patterns and anomalies under community scale. Spatial modeling approaches, including geographically weighted regression and spatial machine learning, allow for the examination of spatially heterogeneous relationships and local drivers. Additionally, geo-visualization techniques facilitate intuitive mapping of circadian disparities. Emerging geospatial big data from wearable devices and social media offer high-resolution, time-stamped insights into human behavior, providing new opportunities to capture and understand the spatial-temporal dynamics of circadian rhythms (Massar et al., 2021; Sinclair et al., 2023).

**Figure 1.**
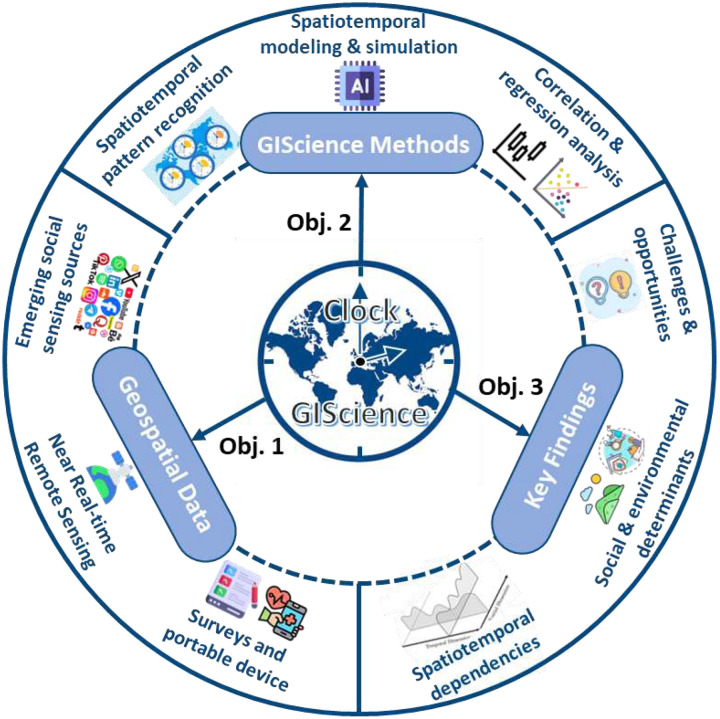
Research objectives of the systematic summary on the applications of GIScience in human circadian rhythm research

Given the growing potential of GIScience in circadian rhythm research, a comprehensive summary of data, methods, findings, challenges, and future directions is needed. This study synthesizes current knowledge on how GIScience contributes to understanding circadian patterns and their relationships with social-environmental factors. It has three objectives (Figure 1): (1) to identify commonly used geospatial data sources and spatial-temporal scales; (2) to categorize GIScience analytical techniques applied in circadian studies; and (3) to summarize empirical findings on social and environmental influences. Accordingly, this investigation addresses four questions: (a) What are the spatial, temporal, and demographic scopes of existing research? (b) What data and methods are used? (c) Which key factors influence circadian rhythms? (d) What challenges and opportunities does GIScience present in this field?

## Methods

This systematic summary comprehensively synthesizes research that focuses on the intersection of circadian rhythm, social-environmental factors, and GIScience. Both quantitative and qualitative studies were included to offer a detailed overview of the significant findings and identify gaps in the existing literature. The systematic summary is conducted by five steps: (1) defining research questions, (2) searching relevant studies, (3) selecting studies based on inclusion criteria, (4) extracting and synthesizing data, and (5) analyzing and interpreting the synthesized data. The rest of this section details each step.

We conducted a systematic literature search across three databases: Web of Science, PubMed, and ScienceDirect. Web of Science provides multidisciplinary coverage, PubMed focuses on biomedical and life sciences, and ScienceDirect hosts scientific and technical publications. Searches were performed in January 2025 to capture studies published up to December 31, 2024. To ensure consistency, the “Title–Abstract–Keywords” search strategy was applied across all three databases to identify relevant literature.

Table 1 summarizes the retrieved literature using different keyword combinations. This structured approach identified 1,633 initial papers including 1,344 research articles, 215 literature reviews, and 74 others such as conference papers and book chapters. The number of identified literatures using combinations of broader keywords (e.g., component 1 keywords + geograph*) is much larger than those using domain-specific keywords (e.g., component 1 keywords + GIS/GeoAI). In the three databases, the total number of circadian rhythm studies using GIS or GeoAI technologies is 0 – 15 while the number of circadian rhythm studies using spatial analysis or geography is 476 – 969.

**Figure 2:**
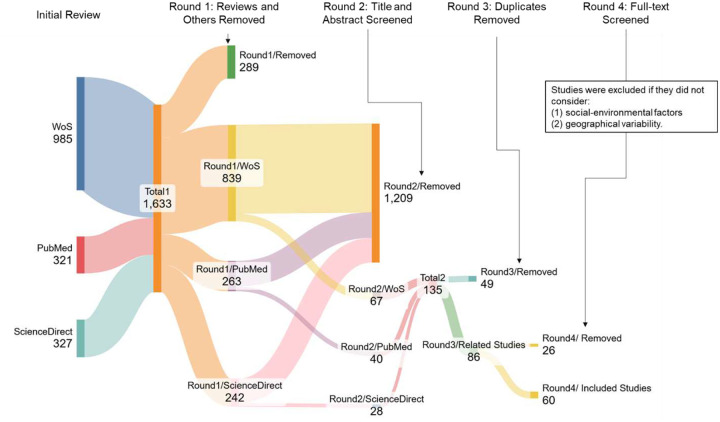
Sankey diagram of the paper selection workflow.

Figure 2 presents a Sankey diagram of the literature selection process. Initial retrieval yielded 985 papers from Web of Science, 321 from PubMed, and 327 from ScienceDirect. After excluding reviews and non-empirical works, 1,344 articles remained. Title and abstract screening removed 1,209 irrelevant studies, leaving 135 articles. Following the removal of 49 duplicates, 86 studies underwent full-text review. An additional 26 were excluded for focusing solely on clinical conditions, lacking social-environmental factors, or not incorporating GIScience approaches. Ultimately, 60 articles met all inclusion criteria and were retained for analysis, ensuring consistency in methodological rigor and relevance to GIScience-based circadian rhythm research.

For answering four research questions in this literature investigation, a structured data extraction framework was developed to collect and analyze key information from each study. As shown in Table 2, the framework includes eight major categories: Publication Information, Research Context, Sample Information, Data Characteristics, Methods, Health Impacts, Major Findings, and Limitations.

Table 2 includes clarified terms for research context. Two key aspects were extracted: (1) the first author’s country/region and (2) the study area location. The first reveals geographic disparities in research production, while the second highlights the spatial scope and potential gaps in studied populations or regions. Spatial scale (e.g., district, city, state, national, global) reflects data availability and helps contextualize circadian patterns within specific environments. Temporal scale describes data collection duration (e.g., daily, seasonal, annual, multi-year) and is essential for interpreting the dynamics and variability of circadian rhythms.

We adopted multiple analytical approaches to synthesize the literature. First, word cloud analysis was used to identify frequently occurring terms in titles and keywords, providing an overview of dominant themes and emerging trends in GIScience-based circadian rhythm research. Second, a co-occurrence bibliometric network was constructed using VOSviewer to examine relationships among studies based on shared metadata, helping identify major research clusters and interdisciplinary patterns. Third, spatial and temporal distributions of studies were visualized to reveal regional disparities and temporal trends. Fourth, we summarized the data sources, categories, and granularities used to study circadian rhythms and associated social-environmental factors. Fifth, analytical methods were classified into spatial and non-spatial approaches, highlighting differences between traditional statistical techniques and spatially explicit models. Finally, geo-visualization mapping was applied to integrate findings and illustrate the spatially heterogeneous effects of social-environmental factors on human circadian rhythms.

## Results

**Figure 3.**
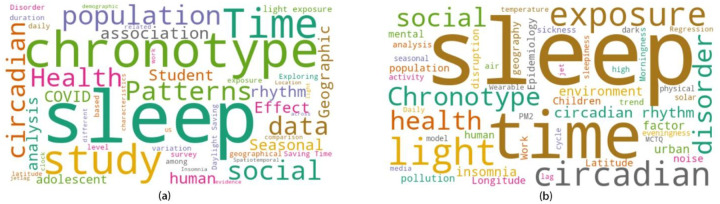
Word clouds derived from (a) titles and (b) keywords of the selected 60 papers.

This literature summary encompasses a total of 60 original peer-reviewed research articles published from 2001 to 2024. These articles were sourced from a diverse range of 8 disciplinary categories based on journals’ metadata, including Public Health (22 papers), Biology (16), Environmental Science (9), Social Science (6), Geography (2), Engineering (2), Psychology (2), and Neuroscience (1). The word clouds derived from titles and keywords of all 60 publications were shown in Figure 3. Words of higher frequency are displayed in larger fonts. Dominating two figures are terms such as “sleep”, “time”, “chronotype” and “circadian”, indicating a significant focus on the temporal changes of human daily behaviors, especially the sleep/wake cycle. The frequency of “spatial”, “geograph*”, “latitude”, “longitude” and “location” appearing in word clouds is low, which means there is a research gap in analyzing the relationship between circadian rhythm and social-environmental factors from geographical perspective. Additionally, terms like “patterns”, “association” and “effects” in the word cloud derived from the title emphasize the research focus on the relationship between social-natural factors and circadian rhythms. The word cloud based on the keywords includes more detailed information about social-environmental factors, such as “light”, “pollution”, “noise”, and “temperature”, underscoring the popular environmental and climatic factors in previous research.

**Figure 4:**
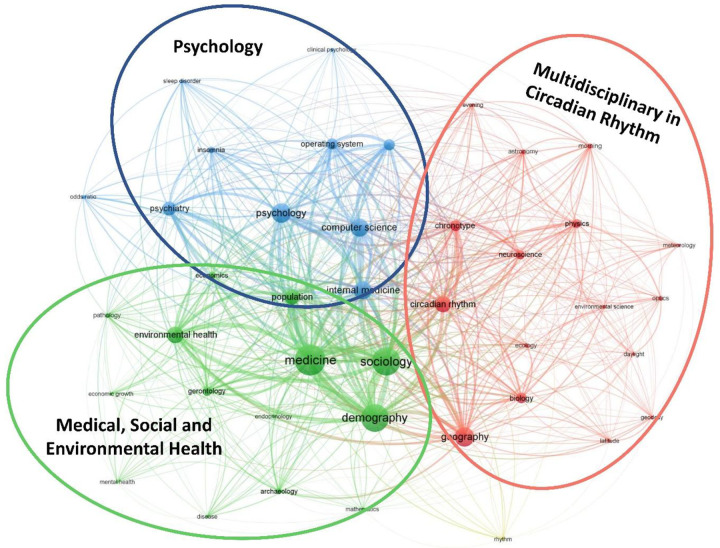
Co-occurrence network and research clusters derived from the abstracts of synthesis articles.

The co-occurrence network generated from abstracts reveals distinct thematic clusters and research priorities in circadian rhythm and GIScience studies (Figure 4). Three clusters of research themes were detected: multidisciplinary in circadian rhythm research (red), medical, social, and environmental health (green), and psychology (blue). The red cluster comprises studies from various disciplines, such as geography, biology, ecology, and neuroscience, reflecting a growing interest in investigating circadian rhythms using multidisciplinary approaches. The green cluster encompasses studies connecting circadian rhythms to public health and societal outcomes, such as environmental health, mental health, and economic well-being. The blue cluster focuses on clinical and psychological dimensions of circadian rhythms, such as sleep disorders, insomnia, and psychiatry, highlighting the medical relevance of circadian research.

**Figure 5:**
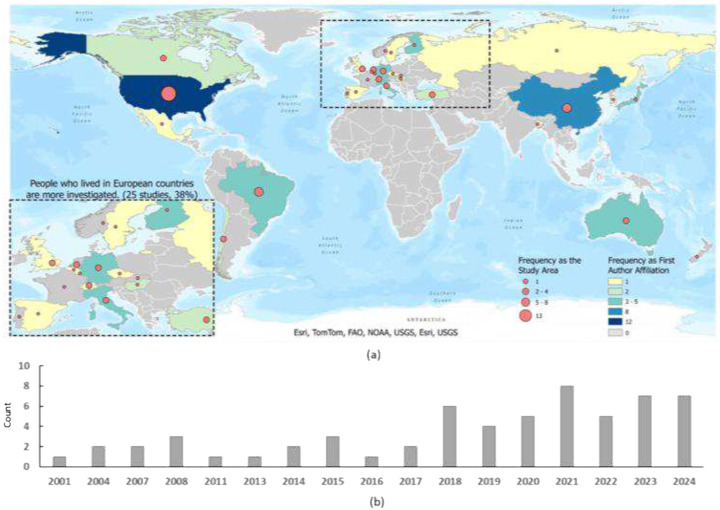
Spatial and temporal distribution of included studies. (a) Global distribution of study areas and first author affiliations. (b) Publication trends from 2001 to 2024.

Figure 5a shows the distribution of study areas and first author affiliations. Scholars from the United States (12 studies, 20%) and China (8, 13%) contribute a large share, while Europe accounts for over 30% (19 studies), led by Germany, Finland, Italy, and others. In contrast, no studies were reported from African-affiliated researchers, highlighting a clear geographic imbalance dominated by the United States, China, and Europe. Study areas are similarly concentrated in Europe (25 studies, 38%), the United States (13, 20%), and China (8, 12%), with limited coverage in Africa. Additionally, publications have increased steadily from 2001 to 2024, with a notable surge after 2018 (Figure 5b), reflecting growing interest in GIScience-based circadian rhythm research.

**Figure 6.**
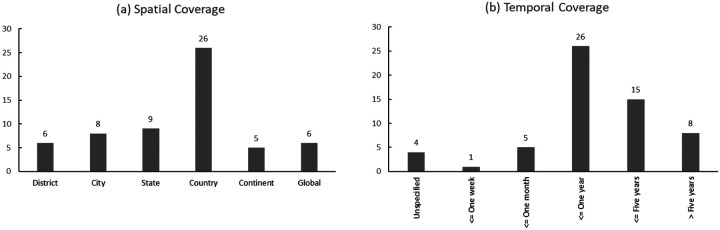
(a) Spatial and (b) temporal coverage of the included studies

Analysis of the spatial and temporal coverage of the included studies reveals distinct patterns in their geographic scope and data duration. The spatial scale of each study was categorized into six levels: district, city, state, country, continent, and global (from smallest to largest). Most studies were conducted at the country level (26), followed by state (9), city (8), global (6), and district (6) scales (Figure 6a). Figure 6b illustrates the temporal coverage of the retrieved studies. A majority (32 studies, 53%) collected data over a period of less than one year, while others spanned 1~4 years (15 studies, 25%) or more than 5 years (8 studies, 13%). This variation across spatial and temporal scales suggests that current research on circadian rhythm disruptions tends to focus on national-scale analyses with short-term observations, underscoring the need for cross-country or localized spatial contexts and longer-term investigations.

**Figure 7.**
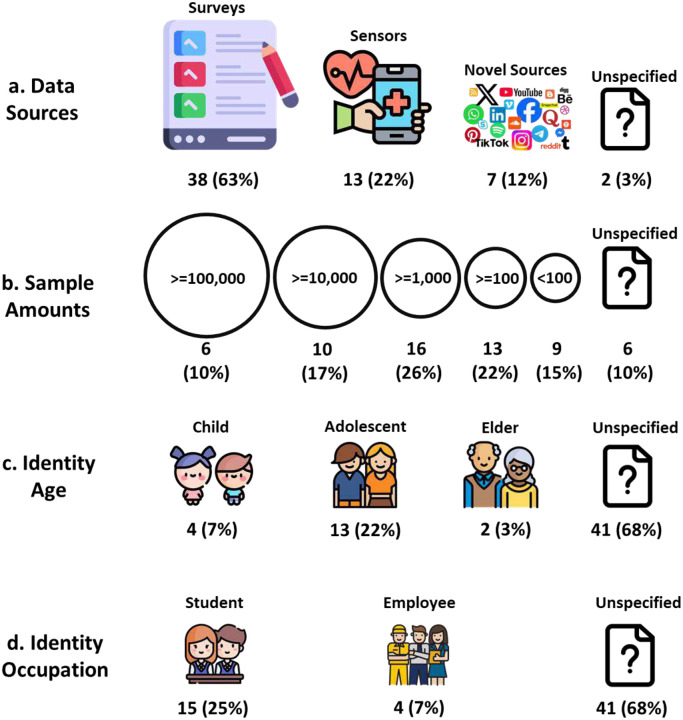
Summary statistics of circadian rhythm data characteristics across included studies, including (a) data sources, (b) sample sizes, (c) participant demographics, and (d) occupational categories.

We extracted information based on the source, the amount, and the identity of samples (Figure 7) to comprehensively understand the characteristics of human circadian rhythm data. Figure 7(a) shows that the majority of circadian rhythm data are derived from surveys (39 studies) and sensors (13 studies). Some studies use novel methods to collect circadian rhythm information, such as social media platforms (3 studies) and websites (4 studies). Figure 7(b) displays that the number of samples in circadian rhythm studies is concentrated in the range 1000 to 10,000, while six studies have sample sizes larger than 100,000. Figures 7(c) and 7(d) show that, compared with other age groups and occupations, adolescents and students are the primary subjects of circadian rhythm research.

**Figure 8.**
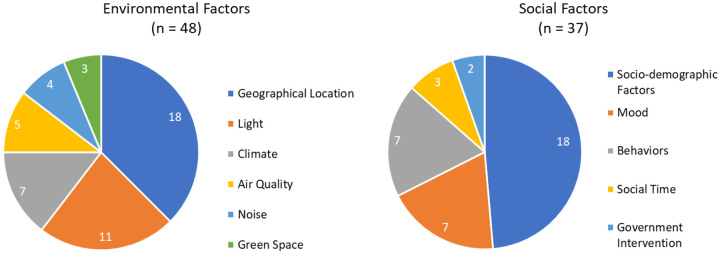
Social-environmental factors associated with human circadian rhythms.

Figure 8 summarizes the environmental and social factors examined in included studies. Overall, more studies focused on environmental factors (48) than social factors (37). Among environmental variables, geographic factors (latitude, longitude, altitude) were most common (38%), followed by light conditions (23%) and climate factors such as temperature and humidity (15%). Other factors included air quality, noise, and green space. In contrast, social-demographic variables (e.g., economy, education, age, gender, urbanization) dominated social factors (49%). Human mood and behavior received moderate attention, while government interventions and social schedules were less frequently examined in circadian rhythm research.

**Figure 9:**
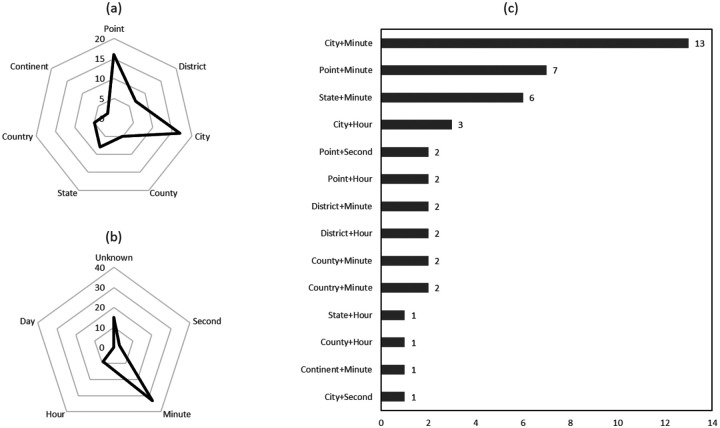
Spatial unit and temporal resolution of data sources

We explored the spatial and temporal granularities of circadian rhythm data to identify common data-collection patterns (Figure 9). Spatial scales varied, with point- and city-level data most frequently used (16 and 17 studies), while coarser units such as states, districts, counties, and continents were less common. Temporal resolutions also differed, with minute-level data dominating (33 studies), followed by hourly (9) and second-level (3) data. The most common spatial–temporal combination was “City + Minute” (13 studies), followed by “Point + Minute” (7), reflecting a strong preference for high-frequency monitoring of circadian rhythms, particularly in urban contexts.

Most studies employed non-spatial methods, including association analysis, causal inference, and dimension reduction techniques (Table 3). Common approaches for preliminary analysis included ANOVA (16 studies), Chi-square tests (6), and correlation analyses (12), used to examine relationships between circadian rhythms and social-environmental factors. Linear regression was the dominant modeling approach (26 studies, 72.2%), explaining the effects of these factors. In contrast, only a small proportion of studies (13.3%) applied spatial methods, such as spatial correlation (5 studies) and spatial causality models (6). Overall, while conventional statistical methods remain prevalent, the limited use of spatial algorithms highlights a gap in capturing geographic heterogeneity and spatial dependence. Expanding spatially explicit approaches would improve understanding of how contextual and locational factors shape circadian rhythm disruptions.

Among the included literature, 8 studies examined location-based associations between social factors and circadian rhythms, including socio-demographics, social schedules, mood, COVID-19 exposure, and human behaviors. For example, Pronk et al. (2024) identified sleep variations across 13 culturally defined regions in the U.S. Moussa-Chamari et al. (2024) found geographic differences in sleep quality among college students, with French students reporting better sleep than those in several other countries. Yan et al. (2022) demonstrated that children in socially vulnerable areas face higher risks of sleep disorders, while Galina et al. (2021) reported poorer sleep among adolescents in highly urbanized environments. Leypunskiy et al. (2018), using geotagged Twitter data, showed that social schedules produce geographically varying patterns of social jet lag. During the COVID-19 pandemic, Al-Mamun et al. (2023) observed higher rates of abnormal sleep in regions with greater exposure based on GIS techonolgies.

In comparison, environmental factors have been more extensively studied, with 48 papers addressing their spatial effects on circadian rhythms. Geolocation plays a key role, with studies examining longitudinal, latitudinal, and altitudinal influences. Masal et al. (2015) found that longitude affects chronotype, with increasing eveningness from east to west. Brockmann et al. (2017) reported longer sleep durations at higher latitudes, while Muñoz et al. (2021) showed increased sleep disturbances among high-altitude workers. Light exposure is another critical factor. Porcheret et al. (2018) found no significant geographic differences in chronotypes based on light exposure, whereas Giuntella and Mazzonna (2019) showed that considering different time zones, extended evening daylight reduces sleep duration and increases insufficient sleep risk. Artificial light at night also contributes to spatial disparities, with Pilz et al. (2018) reporting earlier sleep in less urbanized areas compared to more urban settings. Moreover, the association between outdoor artificial light at night and short sleep duration was stronger in regions with higher poverty levels (Xiao et al., 2020). Temperature and seasonal variation further shape spatial disparities of circadian rhythms disorder. Minor et al. (2022) demonstrated that high temperatures significantly influence afternoon rest patterns, particularly above certain thresholds. Tian et al. (2024) found that extreme heat reduces daytime mobility while increasing evening activity, with notable spatial variability across regions. Other environmental factors, such as noise, air pollution, and green space, are closely tied to build environments and exhibit strong geographic dependence. For instance, Joost et al. (2018) identified spatial clusters of daytime sleepiness linked to nighttime noise exposure. Pelgrims et al. (2021) found associations between black carbon exposure and sleep-related mental health outcomes. Gui et al. (2024) reported regionally heterogeneous effects of air pollution on childhood sleep disorders. In contrast, Astell-Burt et al. (2013) showed that individuals living in greener neighborhoods are less likely to experience short sleep.

**Figure 10.**
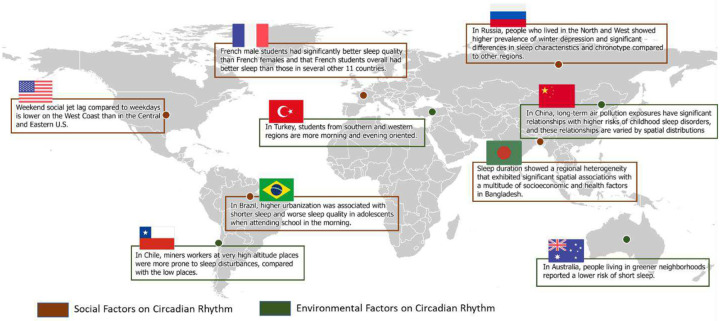
Spatial heterogeneity on human circadian rhythms under various social-environmental contexts

Hence, there are significant studies (as shown in Figure 10) which highlight the importance of both social and environmental contexts in shaping the spatial variability of circadian rhythms, with environmental factors showing broader and more consistent geographic influences.

## Discussion

This literature summary shows that social jetlag, seasonal affective disorder, sleep disturbances, and chronotype exhibit pronounced spatiotemporal heterogeneity rather than uniform population-level patterns. These phenomena are embedded in place-based social and environmental contexts, varying across regions, seasons, and socio-spatial settings. Factors such as latitude, urbanization, work–school schedules, and socioeconomic conditions interact with environmental influences like photoperiod and artificial light exposure, producing geographically uneven circadian misalignment. This heterogeneity challenges aspatial approaches and underscores the importance of Geographic Information Science (GIScience) frameworks that account for spatial scale, dependence, and contextual variability, advancing understanding in health geography.

The investigation also identifies major data gaps in circadian health research, particularly in data diversity, scale, and population coverage. Most studies rely on questionnaire-based surveys, which, while informative, are limited by small sample sizes, coarse temporal resolution, and restricted spatial coverage. Emerging data sources, such as satellite-derived nighttime lights, social media data, wearable device records, and other digital traces, remain underutilized. Additionally, population biases are evident, with a focus on children and adolescents, especially students, while adults, older populations, and working groups are underrepresented. This imbalance limits the generalizability of findings across life stages and social contexts.

Moreover, existing research has primarily focused on a narrow set of determinants, including geographic location, light exposure, climate, and demographic characteristics. Although these studies provide valuable descriptive insights, spatial analytical methods are rarely used to examine spatial dependence, multi-scale variation, or place-specific effects. This methodological gap may reflect several interconnected factors. Circadian health research has traditionally developed within clinical, psychological, and sleep-science disciplines, where individual-level explanatory models and standard statistical techniques are more established than spatial approaches. In addition, limited methodological literacy across disciplinary boundaries may hinder the integration of GIScience methods into circadian health studies. As a result, complex relationships among circadian rhythms, environmental exposures, and socio-spatial contexts are often oversimplified.

Building on identified data gaps and limited spatial methodologies, future circadian health research should adopt more integrative and spatially informed frameworks. First, expanding data diversity is essential by combining traditional surveys with emerging large-scale sources, such as satellite-derived nighttime light, social media data, and wearable device records, to capture behaviors at finer spatial and temporal scales and reduce the magnitude and impact of geo-health uncertainty (Delmelle et al., 2022). Second, studies should move beyond treating social and environmental factors as static, instead applying spatially explicit approaches to examine how their effects vary across locations, scales, and time. Investigating spatial heterogeneity and place-specific mechanisms can deepen understanding of circadian processes. Finally, broadening study populations beyond students to include working adults and older groups will improve generalizability across the life course.

In conclusion, this systematic summary of 60 studies highlights advances in integrating GIScience with circadian rhythm research, revealing significant spatial disparities. Research is concentrated in Europe, the United States, and China, with data primarily derived from surveys and sensors, while emerging geospatial data sources remain underused. Environmental factors such as geographic location and light exposure, along with social factors like income, urbanization, gender, and age, are most frequently examined. However, studies predominantly rely on traditional statistical methods, with limited use of spatial analysis. Overall, findings demonstrate strong spatial and temporal dependencies, with factors such as light, noise, social schedules, and COVID-19 exhibiting notable geographic heterogeneity in circadian disruptions.

## Figures and Tables

**Table 1.** Summary of search keywords and retrieved literature counts by database and keyword combination.

Keywords	Component 1	Circadian, Biological Clock, Chronotype, Sleep/Wake				
	Component 2	geograph*	spatial analysis	GIS	GeoAI	locality, location-based, location-aware
Web of Science	Research Article	458	318	9	0	54
	Literature Review	81	31	0	0	6
	Others	19	8	0	0	1
PubMed	Research Article	245	2	3	0	13
	Literature Review	58	0	0	0	0
Science-Direct	Research Article	73	96	3	0	70
	Literature Review	16	6	0	0	17
	Others	19	15	0	0	12
Total		1633 (Research Article: 1344, Literature Review: 215, others: 74)				

**Table 2:** Information items extracted from reviewed papers for literature analysis.

Category	Information Item	Research Questions
**Publication Information**	Article authors	(a), (b) and (c)
	Article title	
	Publication year	
	Journal name	
	Keywords	
	Abstract	
	Research areas	
**Research Context**	Research objective	(b)
	Country/region of the first author	
	Country/region of the study area	
	Spatial scale of the study	
	Temporal scale of the study	
**Sample Information**	Sample size	(a)
	Sample type or identity	
**Data**	Type of circadian rhythm data	(a) and (b)
	Type of social-environmental data	
	Data sources	
	Spatial and temporal resolutions of data	
**Methods**	Analytical methods used	(a) and (b)
	Methodological innovations	
	Methods type	
**Health Impacts**	Health risks	(c)
	Spatial variation of circadian rhythms	
	Temporal variation of circadian rhythms	
**Major Findings**	Major findings of the study	(d)
**Challenges and Limitations**	Challenges and limitations of the study	(d)

Note: The last column indicates which research questions can be answered by the information under each category; the research questions include (a) What are the spatial, temporal, and demographic scopes of existing research? (b) What data and methods are used? (c) Which key factors influence circadian rhythms? (d) What challenges and opportunities does GIScience present in this field?

**Table 3.** Spatial and non-spatial statistical methods employed in the included studies.

Type	Category	Model Name	Count
Non-Spatial Method (85)	Preliminary Comparisons Analysis (48)	Analysis of Variance (ANOVA)	16
		T-test	8
		Pearson’s Correlation	7
		Chi-squared test	6
		Spearman’s Correlation	5
		Analysis of Covariance (ANCOVA)	4
		Cross-Correlation	2
	Regression Analysis (36)	Linear Regression	26
		Logistic Regression	8
		Locally Weighted Scatterplot Smoothed Curve Regression	1
		Multivariate Temporal Regression	1
	Other Analysis (1)	Principal Component Analysis	1
Spatial Method (13)	Preliminary Comparisons Analysis (5)	Spatial Autocorrelation	4
		Spatial Cluster Analysis	1
	Regression Analysis (6)	Spatial Regression Model	4
		Geographic Weighed Regression	1
		Geographical Convergent Cross Mapping	1
	Other Analysis (2)	Global Cities Cost Model	1
		Gravity Model	1

Note: Among the reviewed studies, 20 papers combined preliminary comparison analysis with regression analysis. In addition, 14 papers employed multiple preliminary comparison analysis methods without regression analysis, whereas 5 papers employed multiple regression analysis methods without preliminary comparison analysis.

## References

[R1] Al-Mamun F., Rahman M. M., Islam S. M. S., and Hossain M. S. 2023. Sleep duration during the COVID-19 pandemic in Bangladesh: A GIS-based large sample survey study. Scientific Reports 13(1): 3368. 36849735 10.1038/s41598-023-30023-1 PMC9969935

[R2] Astell-Burt T., Feng X., and Kolt G. S. 2013. Does access to neighbourhood green space promote a healthy duration of sleep? Novel findings from a cross-sectional study of 259,319 Australians. BMJ Open 3(8): e003094. 10.1136/bmjopen-2013-003094 PMC3740246 23943772

[R3] Brockmann P. E., Diaz B., Damiani F., Villarroel L., Núñez F., and Bruni O. 2017. Geographic latitude and sleep duration: A population-based survey from the Tropic of Capricorn to the Antarctic Circle. Chronobiology International 34(3): 373–381. 28128998 10.1080/07420528.2016.1277735

[R4] Czeisler C. A., Duffy J. F., Shanahan T. L., Brown E. N., Mitchell J. F., Rimmer D. W., Ronda J. M., Silva E. J., Allan J. S., Emens J. S., and Dijk D. J. 1999. Stability, precision, and near-24-hour period of the human circadian pacemaker. Science 284(5423): 2177–2181. 10381883 10.1126/science.284.5423.2177

[R5] Delmelle E.M., Desjardins M.R., Jung P., Owusu C., Lan Y., Hohl A. and Dony C., 2022. Uncertainty in geospatial health: challenges and opportunities ahead. Annals of epidemiology, 65, pp.15–30. 34656750 10.1016/j.annepidem.2021.10.002

[R6] Galina S. D., Pereira E. F., Teixeira C. S., and Lopes A. S. 2021. Daily light exposure, sleep–wake cycle and attention in adolescents from different urban contexts. Sleep Medicine 81: 410–417. 33826994 10.1016/j.sleep.2021.03.012

[R7] Gooley J. J., Chamberlain K., Smith K. A., Khalsa S. B. S., Rajaratnam S. M. W., Van Reen E., Zeitzer J. M., Czeisler C. A., and Lockley S. W. 2011. Exposure to room light before bedtime suppresses melatonin onset and shortens melatonin duration in humans. Journal of Clinical Endocrinology & Metabolism 96(3): E463–E472. 21193540 10.1210/jc.2010-2098 PMC3047226

[R8] Giuntella O., and Mazzonna F. 2019. Sunset time and the economic effects of social jetlag: Evidence from US time zone borders. Journal of Health Economics 65: 210–226. 31030116 10.1016/j.jhealeco.2019.03.007

[R9] Gui Z.-H., Zhang X., Liu Y., and Wang J. 2024. Exposures to particulate matters and childhood sleep disorders—A large study in three provinces in China. Environment International 190: 108841. 38917626 10.1016/j.envint.2024.108841

[R10] Joost S., Matz C. J., Bucher B., and Vienneau D. 2018. Spatial clusters of daytime sleepiness and association with nighttime noise levels in a Swiss general population (GeoHypnoLaus). International Journal of Hygiene and Environmental Health 221(6): 951–957. 29861399 10.1016/j.ijheh.2018.05.004

[R11] Leypunskiy E., Kıcıman E., Shah M., Walch O.J., Rzhetsky A., Dinner A.R. and Rust M.J., 2018. Geographically resolved rhythms in Twitter use reveal social pressures on daily activity patterns. Current Biology, 28(23), pp.3763–3775. 30449672 10.1016/j.cub.2018.10.016 PMC6590897

[R12] Luan H. and Law J., 2014. Web GIS-based public health surveillance systems: a systematic review. ISPRS International Journal of Geo-Information, 3(2), pp.481–506.

[R13] Martins A. J., Lima-Costa M. F., and Peixoto S. V. 2020. The effect of urbanization on sleep, sleep/wake routine, and metabolic health of residents in the Amazon region of Brazil. Chronobiology International 37(9–10): 1335–1343. 32777972 10.1080/07420528.2020.1802287

[R14] Masal E., Aydin A., and Ertan T. 2015. Effects of longitude, latitude and social factors on chronotype in Turkish students. Personality and Individual Differences 86: 73–81.

[R15] Massar S.A., Chua X.Y., Soon C.S., Ng A.S., Ong J.L., Chee N.I., Lee T.S., Ghosh A. and Chee M.W. 2021. Trait-like nocturnal sleep behavior identified by combining wearable, phone-use, and self-report data. NPJ digital medicine 4(1): 90. 34079043 10.1038/s41746-021-00466-9 PMC8172635

[R16] Mills J. N. 1966. Human circadian rhythms. Physiological Reviews 46(1): 128–171. 5323500 10.1152/physrev.1966.46.1.128

[R17] Minor K., Bjerre-Nielsen A., Jonasdottir S. S., Lehmann S., & Obradovich N. 2022. Ambient heat and human sleep. One Earth, 5(1), 52–63.

[R18] Moussa-Chamari I., Farooq A., Romdhani M., Washif J. A., Bakare U., Helmy M., Al-Horani R. A., Salamh P., Robin N., and Hue O. 2024. Exploring sleep patterns in 3,475 college students: A comparative study of geographical location, gender, and age. Sleep Science 18(2): e128–e137. 40672899 10.1055/s-0044-1788288 PMC12263208

[R19] Muñoz S., Nazzal C., Jimenez D., Frenz P., Flores P., Alcantara-Zapata D. and Marchetti N. 2021. Health effects of chronic intermittent hypoxia at a high altitude among Chilean miners: rationale, design, and baseline results of a longitudinal study. Annals of Work Exposures and Health, 65(8): 908–918. 34435202 10.1093/annweh/wxab029

[R20] Pelgrims I., Lefebvre W., Vanpoucke C., and Meysman F. J. R. 2021. Association between urban environment and mental health in Brussels, Belgium. BMC Public Health 21(1): 635. 33794817 10.1186/s12889-021-10557-7 PMC8015067

[R21] Pilz L. K., Carissimi A., Oliveira M. A., and Benedito-Silva A. A. 2018. Sleep and light exposure across different levels of urbanisation in Brazilian communities. Scientific Reports 8(1): 11389. 30061685 10.1038/s41598-018-29494-4 PMC6065379

[R22] Porcheret K., Wald L., and Noseda J. 2018. Chronotype and environmental light exposure in a student population. Chronobiology International 35(10): 1365–1374. 29913073 10.1080/07420528.2018.1482556 PMC6234547

[R23] Pronk N. P., Arena R., Laddu D., and Woodard C. 2024. Regional cultures and insufficient sleep in the United States. Journal of Activity, Sedentary and Sleep Behaviors 3: 4. 40217336 10.1186/s44167-023-00043-3 PMC11960212

[R24] Roenneberg T., Kumar C. J., and Merrow M. 2007. The human circadian clock entrains to sun time. Current Biology 17(2): R44–R45. 17240323 10.1016/j.cub.2006.12.011

[R25] Rust M.J., Golden S.S. and O’Shea E.K., 2011. Light-driven changes in energy metabolism directly entrain the cyanobacterial circadian oscillator. Science, 331(6014), pp.220–223. 21233390 10.1126/science.1197243 PMC3309039

[R26] Sinclair M., Maadi S., Zhao Q., Hong J., Ghermandi A. and Bailey N., 2023. Assessing the socio-demographic representativeness of mobile phone application data. Applied geography, 158, p.102997.

[R27] Tian H., Cai H., Hu L., Qiang Y., Zhou B., Yang M., and Lin B. 2024. Unveiling community adaptations to extreme heat events using mobile phone location data. Journal of Environmental Management 366: 121665. 39032252 10.1016/j.jenvman.2024.121665

[R28] Xiao Q., Gee G., Jones R.R., Jia P., James P. and Hale L., 2020. Cross-sectional association between outdoor artificial light at night and sleep duration in middle-to-older aged adults: the NIH-AARP Diet and Health Study. Environmental research, 180, p.108823. 31627155 10.1016/j.envres.2019.108823 PMC6996197

[R29] Yan F., Xu Z., and Zhao Y. 2022. Examining associations between neighborhood-level social vulnerability and care for children with sleep-disordered breathing. Otolaryngology–Head and Neck Surgery 166(6): 1118–1126. 35259035 10.1177/01945998221084203 PMC9531179

